# Evolutionary Perspective in Rickets and Vitamin D

**DOI:** 10.3389/fendo.2019.00306

**Published:** 2019-05-15

**Authors:** Ze'ev Hochberg, Irit Hochberg

**Affiliations:** ^1^Rappaport Family Faculty of Medicine, Technion – Israel Institute of Technology, Haifa, Israel; ^2^Institutes of Endocrinology, Diabetes and Metabolism, Rambam Health Care Campus, Haifa, Israel

**Keywords:** rickets, vitamin D, skin color, MC1R, evolution

## Abstract

Modern lifestyle limits our exposure to sunlight, which photosynthesizes vitamin D in the skin, and the incidence of nutritional rickets has been resurging. Vitamin D is one of the first hormones; it is photosynthesized in all organism from the phytoplankton to mammals. A selective sweep of the promoter of the vitamin D receptor (VDR) happened as soon as *Homo sapiens* migrated out of Africa; it co-adapted with skin color genes to provide adaptation to latitudes and the levels of exposure to ultraviolet (UV)B radiation along the route out of Africa. Exposure to UVB radiation balances the need for vitamin D photosynthesis and degradation of folic acid by UVB radiation. Skin color follows a latitude distribution: the darkest populations dwell in the tropical belt; and the fair-skinned populations inhabit the northern countries. Due to their greater need for calcium during their reproductive life, the skin color of women is lighter- than that of men. Vitamin D is essential for mineral homeostasis and has a wide variety of non-skeletal functions, of which the most important for natural selection is a regulatory function in the innate immune system. In the human fossil record, vitamin D deficiency coincided with bone tuberculosis. About 6,000 years ago, a diet which included cow's milk provided Neolithic humans with twice as much calcium and was more alkaline than that of its Paleolithic predecessors. Adiposity is negatively associated with the vitamin D status and obese individuals require 2–3 times more vitamin D than non-obese individuals to normalize circulating 25OHD levels. In an era of an obesity epidemic, we need more research to determine whether adiposity should be considered when determining the dietary requirements for vitamin D and calcium and the optimal serum 25OHD levels.

## PROLOG

Rickets and osteomalacia have always been present in European populations and people from the Middle East, as evidenced from paleopathological specimens of juvenile rachitic bones and teeth with dentin defects which have been found in various archeological sites. These specimens reveal a high prevalence of mineralization disruption in our human ancestors who lived in the subtropical and temperate climatic zones from over 100,000 years ago to the present era (1). Although one can only speculate on the exact cause of rickets or osteomalacia in these specimens, vitamin D deficiency may have been the cause.

In 2008, Kappelman et al. reported the notable circumstances which surrounds the discovery of the first fossil hominin calvaria from Turkey (2). This specimen, attributed to *Homo erectus*, preserves unusual findings on the endocranial surface of the frontal bone that is consistent with a diagnosis of *Leptomeningitis tuberculosa* (TB). This preserved pathology in a human fossil is the most ancient example of this disease. Kappelman et al. posited that TB was exacerbated in this dark-skinned individual who lived in the northern latitudes by a vitamin D deficiency because of lower levels of ultraviolet radiation (UVR). The presumption of these paleoanthropologists implies the existence of an association between vitamin D deficiency and TB. This presumption also implies that climate was an adaptive challenge to our ancestors during their migration into the temperate regions of Europe and Asia.

At the start of the industrial revolution, rickets, “the English disease,” was estimated to be present in n 50–80% of children in industrialized northern Europe (3), and TB was common (4). In England, the 1840s were known as the hungry forties because many children were literally starving on the streets of London. This description is reinforced by the portrayal of Tiny Tim in Charles Dickens' novel “A Christmas Tale” which was published in 1843. Dickens describes Tiny Tim as having a *short stature, asymmetric crippling, and curious intermittent weakness that would lead to his death, if untreated* (4), and this description strongly suggests that Tiny Tim suffered from a combination of TB and rickets.

In 1849, Williams reported the results of administering fish liver oil (vitamin D) to 234 patients with TB (5). He noted that *even in a few days…the appetite, flesh and strength were gradually improved*“ and concluded that ”*the pure fresh oil from the liver of the cod is more beneficial in the treatment of pulmonary consumption than any agent, medicinal, dietetic, or regiminal, that has yet been employed*“. Tiny Tim did not die prematurely despite fish liver oil not being included in his diet.

In contemporary humans, vitamin D deficiency is highly prevalent globally and vitamin D insufficiency affects many people in many countries. Rickets has become an endemic disease of modern civilization and is most prevalent in children with a dark complexion (6). Modern day vitamin D deficiency/insufficiency is probably due to a mismatch between our genes and our present environment, and an insufficient exposure to sunlight. The reduction in sun exposure is caused by spending many hours indoors at school and in leisure activities; cultural reasons related to clothing; and public health recommendations to avoid sun exposure so as to prevent skin cancers and skin damage (7).

## Evolutionary Perspective

Most plants and animals that are exposed to sunlight have the capacity to make vitamin D, the sunshine hormone. Vitamin D is an ancient hormone: it is photosynthesized in many life forms which range from the early life forms the phytoplankton of 750 million years ago (m.y.a) to present-day mammals (8, 9). Holick et al. (10) reported that *Emiliania huxlei*, a unicellular eukaryotic phytoplankton which exists unchanged for over 750 million years in the Sargasso Sea (a region of the North Atlantic Ocean), can convert 7-dehydrocholesterol to ergosterol. Moreover, this microorganism can suppress the production of ergosterol and induce the generation of provitamin D2 when exposed to UVR.

Although it is understandable why terrestrial animals with a calcified skeleton and lay eggs with a calcified shell need provitamin D for calcium and bone metabolism, the needs and functions of vitamin D in either phytoplankton or zooplankton remain unknown. Holick et al. suggested that provitamin D evolved to protect UVR-sensitive macromolecules from solar UV damage or in regulating membrane permeability to cations, such as calcium (10).

Fish have the utmost natural content of vitamin D (11) because they consume plankton which is rich in vitamin D and is the basis for the entire marine food web. Microalgae contain both vitamin D3 and provitamin D3, 7-dehydrocholesterol, and vitamin D2 is produced in fungi and yeasts when provitamin D2 is exposed to UVB radiation (12). Vitamin D3 and its provitamin, have also been identified in the leaves of several species which mostly belong to the *Solanaceae* family of trees, shrubs, and herbs including leaves but not fruit of potato, tomato, eggplant, and peppers) (13). Humans routinely consume the fruit and roots but not the leaves of these plants (14). UVB irradiation of several plants has been shown to induce the production of flavonols that promote growth (shoot length and fresh weight), and possibly also promote nodulation in the roots of pea plants (15). The plant leaves, roots, and oils which are commonly consumed by humans are being investigated as potential vitamin D sources.

Vitamin D3, which is synthesized in the skin, requires sequential hydroxylations in the liver and kidney to be converted to its biologically active form, 1α,25-dihydroxyvitamin D3 (1,25D3), which binds a unique vitamin D receptor (VDR). 1,25D3 and the VDR are important for calcium absorption, and skeletal development and mineralization, but also for the regulation of proliferation of many cell types (16–18).

Although the VDR, which belongs to the superfamily of nuclear hormones receptors, is well-conserved from *Xenopus* to mammals (19), there has been extensive evolution of the VDR in vertebrates (20). Using a genomic approach, it has been suggested that VDR might be the original nuclear receptor (21). When compared in detail across a series of contemporary vertebrate species, ligand selectivity of vitamin D derivatives for the VDRs are reported to be similar (21). VDR polymorphisms are associated with bone size (22) and the risk and incidence of fractures (23). The widespread abundance of the VDR may be related to a host of recent reports which claim that vitamin D functions as a regulator in many biological processes, which include cell differentiation and proliferation, immunity, muscle strength, and blood pressure control (24).

## Vitamin D and the evolution of skin color in *H. sapiens*

Evolutionary pressures due to variation in climate play an important role in determining phenotypic diversity among and within species. In 1833, Gloger published what has become known as “*Gloger's rule”* on the coloration of birds (25): Within a species of endotherms, the heavily pigmented forms tend to be found in equatorial areas of the globe because of the selective pressures of heat, humidity, and UVR.

The original hominines, who inhabited current-day tropical Africa, required minimal substrate and storage of vitamin D in this sun-rich environment because provitamin D is easily photo-isomerized to biologically active isomers under exposure to high UVB radiation. When these hominines began to migrate to regions which were either north or south of the equator, dark or black pigmentation of the skin became a liability because of shorter lengths of daylight and an increase in the number of sunless days. Inbreeding within white-skinned groups, which continually heightened fair skin, made the development of this new human trait possible. Evolutionary analyses indicate that dark skin was the ancestral trait for modern humans, consistent with the evolution of the *homo* lineage in Africa in regions of high UV intensity (26). Hominid migration and the change of latitude heavily impacted the evolution of skin color genes and the VDR. Originated by Murray in 1934 (27), and then modified by Loomis in 1967 (28), the 'vitamin D hypothesis' was refined by Jablonski and Chaplin (26, 29) and more recently by Tiosano et al. (30). This hypothesis is based on the observation that the skin color of the world's indigenous peoples trails a latitude distribution: the populations with the darkest skin color inhabit the equatorial and tropical belts; the most fair-skinned populations inhabit the northern countries; and those with intermediate pigmentation of their skin inhabit the middle latitudes (26, 29–31).

Exposures to sunlight and UVB radiation have to balance the need for vitamin D photosynthesis, and additional vitamin D-independent effects of UV radiation on immune function [reviewed in (32)] on one hand, and degradation of folic acid by UVR on the other hand. This balance is maintained by melanism, which determines skin color. Based on reflectance measurements, a comprehensive compilation of skin colors of indigenous peoples is now available, and this compilation reveals a strong correlation between skin color and the geographical latitude of the habitat (26). Variation in human skin pigmentation is due to the quantity of melanin, the size of melanin particles, and the distribution of eumelanin (dark melanin) and phaeomelanin (red/yellow melanin) that are generated by the melanocyte. Dark melanin absorbs and scatters the UVB radiation which catalyzes vitamin D3 synthesis. In general, a high amount of dark melanin in the skin slows cutaneous synthesis of vitamin D3. Dark-skinned individuals require a six-time longer exposure to sunlight than fair-skin individuals to achieve the same vitamin D serum levels.

The skin pigmentation of the chimpanzee, which lives in the dark forest, is paler than that of equatorial and Middle Eastern humans. When hominines left the forest for the sun-exposed savannah, they lost their fur, acquired a sweating mechanism, and their skin became pigmented to protect them from the higher levels of UVR. When *H. sapiens* migrated out of Africa, they received significantly less UVB radiation, and their skin depigmented to a degree that permitted UVB-induced synthesis of provitamin D3. Since the skin color of humans correlates with the geographical latitude and the levels of UVR of their habitat (26, 30, 33–35), this correlation is a compromise on the need for vitamin D and the detrimental effect of UVR on folic acid generation (36). Evolution has used the polygenic trait of skin pigmentation as a tool for such balance.

The result of a genome-wide association study of natural hair and skin color in more than 10,000 men and women of European ancestry from the United States and Australia identified several genes that are decidedly associated with skin color, which include *TYR, TYRP1, OCA2, SLC45A2, SLC24A5, KITLG*, and *MC1R* (37–40). In a recent study of 751 subjects with diverse skin colors from a broad range of latitudes, we investigated possible multilocus correlation variation of skin color genes with the VDR (30). We discovered two multilocus networks which involved the VDR promoter and skin color genes and show strong latitudinal clines, even though many of their single gene components do not. Considered one by one, the VDR components of these networks show diverse patterns: no cline, a weak declining latitudinal cline outside of Africa, and a strong in-vs.-out of Africa frequency pattern (30). These results suggested that (1) a selective sweep which favored the VDR promoter haplotype happened as soon as *H. sapiens* migrated out of Africa; (2) the VDR promoter haplotype co-adapted with single nucleotide polymorphisms in the skin color genes; (3) the main skin color gene that correlates strongly with latitude is the melanoma-associated SLC45A2 gene; and (4) the cluster of the VDR promoter with the skin color genes provides a fine-scale adaptation to northern latitudes and decreasing UVB along the route out of Africa.

The strongest determinant of skin color is the cell-surface melanocortin type-1 receptor (MC1R), a G-protein-coupled receptor which is involved in the synthesis of melanin in the melanocyte. Several variant MC1R alleles are associated with the typical Nordic red hair, fair-skinn trait (40, 41). The results of a systematic functional analysis of nine common MC1R variants by *in vitro* expression studies discovered receptors with normal to reduced expression and cyclic AMP coupling (42). Beaumont et al. performed a systematic functional analysis of nine common MC1R variants and correlated these results with the power of the genetic association of each variant allele with pigmentation phenotypes. They found parallels between biological function of several MC1R gene variants and their effect on human pigmentation (42). The common ancestral form of the human *MC1R* gene dates back to 850 k.y.a., and spread out of Africa during the Acheulean expansion which occurred 800 k.y.a. (43). The MC1R gene displays a significant molecular signature of selection within sub-Saharan African populations of purifying selection to maintain its protein structure (40). The selective sweep for gene variants' frequency in a population that define lighter skin in northern latitudes is among the strongest signals of recent selection in humans, with point estimates of selection of 2–10% per generation (44). This rapid pace supports a significant evolutionary advantage of lighter skin as migration advanced to northern latitudes. Although evolution of skin color is partially explained by positive selection of light skin color by mate choice (44), the main disadvantage is attributed to the vitamin D deficiency in dark-skinned humans who live in areas with low levels of UVR.

Another notable feature of human skin color is that the skin color of women, tends to be lighter than that of men (45). This is probably directed by evolutional selection, as the need for calcium is much higher in women due to their periods of pregnancy and lactation, and a fair skin enables women to augment vitamin D generation for a given dose of UVB radiation. Other evolutionary biologists argue that the skin color of women is a result of sexual selection: men prefer women with a light skin color (46).

## Evolutionary Selection Pressure of Vitamin D Deficiency in *H. Sapiens*

Both calcemic and non-calcemic actions of vitamin D have been proposed as explanations for the strong negative evolutionary selection pressures against individuals with vitamin D deficiency. Significant deficiency in vitamin D can lead to dysregulation of calcium absorption and bone metabolism. Severe deficiency can lead to life-threatening hypocalcemia, which in turn may lead to cardiac arrhythmia. Childhood rickets leads to severe skeletal deformities which confer a significant disability with consequent reproductive and survival compromise. In adults, vitamin D deficiency-induced osteomalacia increases the risk of fracture, especially when associated with muscle weakness, another consequence of vitamin D deficiency (47). The results of several studies of contemporary women and newborns in developing countries have identified vitamin D deficiency as a risk factor for adverse pregnancy outcomes, both maternal (pre-eclampsia, infection, and cesarean section delivery) and neonatal (small size for gestational age, low birth weight, and stunting) (48). The excess rate of cesarean sections in women with vitamin D insufficiency may possibly be attributed to pelvic inlet deformities caused by osteomalacia or muscle weakness (49).

The observation that the vitamin D-metabolizing enzymes and the VDR are expressed in cells other than those of the bone, intestine, kidney, and parathyroid gland led to the recognition of the non-calcemic actions of vitamin D. Work done at the turn of the 21st century revealed that vitamin D has a wide variety of non-calcemic functions (8, 10), of which the most important is probably its regulatory function in the innate immune system (50, 51). Accordingly, it is tempting to speculate that this regulatory function has made vitamin D such an extraordinary highlight of human evolution (52). The results of previous investigations revealed that (a) monocytes which were incubated with vitamin D (a) induced anti-tuberculosis activity, and (b) monocytes which were incubated with interferon-gamma developed 25-hydroxyvitamin D3-1-hydroxylase activity (53, 54). The innate antimicrobial defense system uses activation of Toll-like receptors (TLR) to generate 25OHD3-1α-hydroxylase for converting an inactive metabolite into active 1,25D3, which in turn triggers the generation of cathelicidin, which kills *Mycobacterium tuberculosis* (51). It has also been reported that dark-skinned African Americans have lower circulating levels of 25OHD3, they display smaller induction of cathelicidin mRNA, and are more susceptible to *M. tuberculosis* than Caucasian Americans (55). In a study of patients with hereditary vitamin D–resistant rickets (HVDRR), who have a defective VDR, Tiosano et al. reported that cathelicidin expression was lower in monocytes which were collected from individuals with HVDRR than in control monocytes (56). Tiosano et al. also reported that 25OHD3 increased significantly the expression of cathelicidin and VDR in the control monocytes but only slightly in HVDRR monocytes and suppressed TLR2 only in the control monocytes. In another study, Yuk et al. reported that vitamin D3 also induces autophagy in human monocytes/macrophages via cathelicidin (57), and the cytocidal action of neutrophils from patients with HVDRR *on Candida albicans* is defective (58). To assess the clinical relevance of these *in vitro* investigations, 192 healthy adult TB contacts were randomized to obtain a single oral dose of 2.5 mg vitamin D or placebo and followed for 6 weeks in a double-blind controlled trial (59). The viability of *M. tuberculosis* in the two groups was measured using a reporter-gene tagged TB specific antigen, and the investigators found that blood of those individuals who had received vitamin D supplementation reduced the viability of *M. tuberculosis*.

The results of several randomized controlled trials found the prevalence of respiratory infections is lowered by vitamin D supplements in children and adults with vitamin D deficiency (60). In a cohort of newborns of HIV-positive mothers in Tanzania, the authors reported that a low vitamin D intake was associated with a risk of mother-to-child HIV transmission and a high risk of 2-year infant mortality (61).

Clear evidence of evolutionary selection by low vitamin D availability through modulation of immune activity can be demonstrated in the enrichment of VDR binding in lymphoblastoid cell lines from European and Asian populations, but not from an African Yoruba population (62).

## Dietary Calcium Considerations

**Figure 1:**
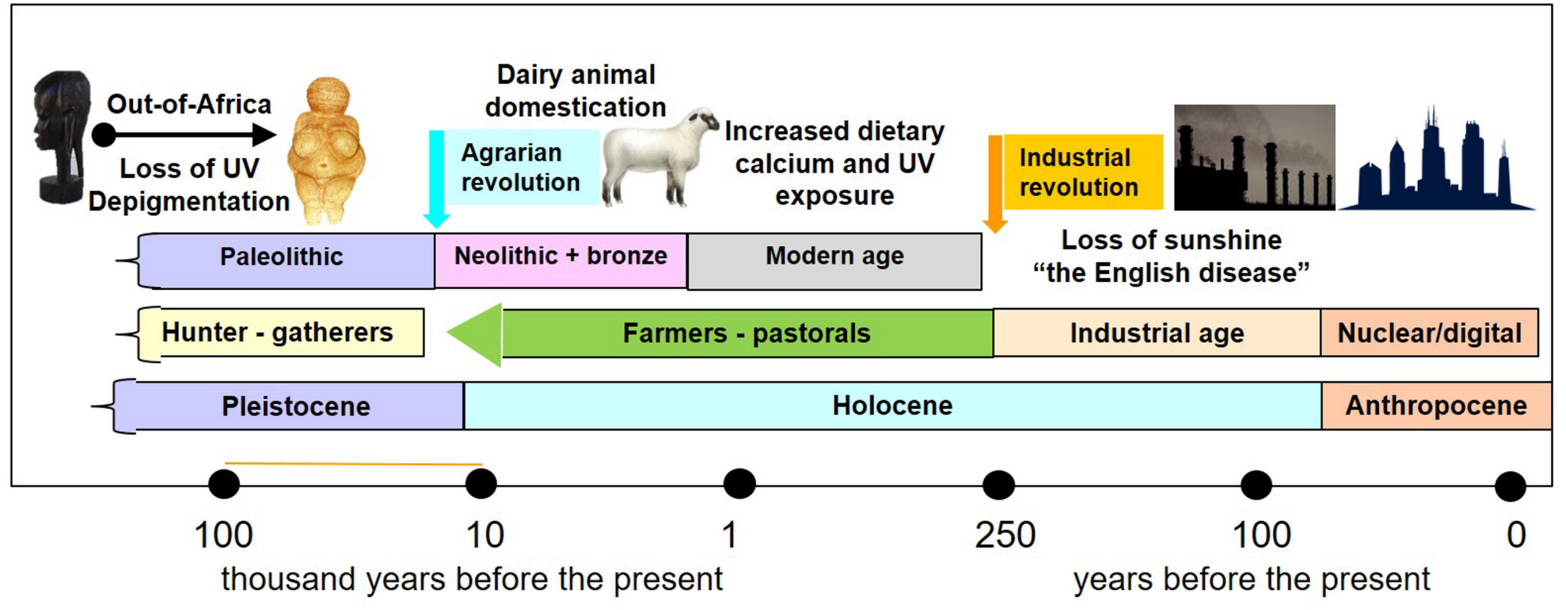
Timeline of geological epochs, archeological periods in human prehistory and history, and the effects of sunshine and dietary calcium. The migration of man out of Africa to northern and southern latitudes 60–130 k.y.a was associated with depigmentation. Transition from the geological Pleistocene epoch to the Holocene epoch coincided with the “agrarian revolution” 10–12,000 years ago and from the archeological Paleolithic period (hunter gatherer tool makers) to the Neolithic farmers. The agrarian revolution happened at different times in different parts of the world, and wherever it happened, it was associated with an increase in dietary calcium and crowding of people in cities. The industrial revolution, which began in Europe in the second half of the 18 century, was associated with rickets – “the English disease” due to industrial air pollution. The transition to the nuclear age, also called the digital age, define also the transition to the Anthropocene epoch, which is associated with further diminution of UVR exposure in humans.

The transition from a lifestyle of hunting and gathering to one of animal and plant domestication 12-8 k.y.a. occurred in parallel or during to the transition from the Pleistocene to the Holocene after the last glacial period (Figure 1). The ecological changes associated with this geological transition led to the agrarian revolution of dairy animal and plant domestication and the transition from the Paleolithic to the Neolithic periods. Although post-weaning lactose intolerance occurs in most mammals, at some point in history human populations in northern Europe adapted to permit the consumption of milk beyond childhood and in adulthood by selecting for persistence of intestinal lactase activity beyond infancy (63). About 6,000 years ago, a diet which included cow's milk provided Neolithic humans with twice as much calcium than that of their Paleolithic predecessors (64) (Table 1). In Western countries, milk is a major food source for calcium, generally providing 36–70% of the dietary calcium (65).

**Table 1:** A comparison of minerals in a Paleolithic diet based on lean meat, fish, fruits, vegetables, root vegetables, eggs and nuts; and a diabetes diet designed in accordance with dietary guidelines (64).

	**Paleolithic diet**	**Diabetes diet**	***P***
Phosphorus (mg)	1233 ± 247	1437 ± 208	0.02
Potassium (mg)	3669 ± 982	3181 ± 908	0.0497
Calcium (mg)	356 ± 102	698 ± 220	0.00002
Magnesium (mg)	307 ± 84	311 ± 68	0.9

The results of calcium balance studies have suggested that man can adapt to relatively minute calcium intake by increasing calcium absorption and decreasing urinary excretion when vitamin D is supplied (66). Using evolutionary logic, this finding suggests that modern people, like primates and hominine ancestors, are capable of adjusting to changing dietary calcium. On the other hand, cereals and legumes, which became major energy sources in agrarian society, are rich in the anti-nutrients, phytate and oxalate, which diminish calcium absorption (67).

The bottleneck of calcium requirements, and apparently a leading evolutionary trigger, is the calcium requirement for skeletal growth in all vertebrate offspring and even more so, in nursing mothers. The calcium that is required for milk production is generated by a dramatic increase in the rates of bone resorption and a decrease in renal calcium excretion (68).

Whatever the physiological consideration, Thacher et al. reported the results of a randomized, double-blind, controlled trial study of 123 Nigerian children with rickets (69). They found that that the intake of calcium in these children was low and that the response to treatment with calcium alone or in combination with vitamin D was better than that to treatment with vitamin D alone. Calcium-deficient rickets shows its presenting signs in toddlers, as compared to vitamin D deficiency which becomes evident in infancy. We have also reported that calcium deficiency is present in severely rachitic toddlers from Egypt (70). Thacher et al. published a systematic review of articles that were published in the last 20 years on the prevalence of nutritional rickets in various geographical regions (71). They found that calcium deficiency is the major cause of rickets in Africa and some parts of tropical Asia, and the prevalence of rickets is increasing in other parts of the world (71).

## The Role of Culture and Technology

Humans have been able to successfully inhabit almost all of the earth's natural environments. Similar to all other organisms, we are still motivated by primordial instincts, among them the drive to propagate our DNA to the limits of possibility (72). DNA is clearly important for the inheritance of traits, but any process or activity which contributes to parent-offspring resemblance within populations has potential evolutionary relevance. These processes and activities, which include culture, technology, customs, traditions, religion, and governance among others, are bequeathed to future generations and impact strongly on human traits and behavior. In addition to biological evolution, we are already relying on culture and technology to retain reproductive fitness. For humans, a culture-determined way of life can be (and has been) reinvented, modified, and changed in accordance with the prevailing climatic and environmental changes. For example, domestication of dairy animals liberated humans 6,000 years ago from the constant search for food, including a source of calcium, and this knowledge was passed down to their descendants. Other examples are the scientific revolution 300 years ago, the industrial revolution at the turn to the 19th century, and the current digital revolution; these revolutions changed our environment and lifestyle at an unprecedented pace as compared to the slow pace of DNA-dependent evolution (72).

Focusing on vitamin D and rickets, we should also include culture and technology in the evolutionary perspective of rickets and vitamin D (73). Culture and technology have significant impacts on habitability of northern and southern latitudes, housing, clothing, work practices, diet, and environmental conditions. The lifestyle of the digital age limits our exposure to sunlight, and as a result, there is a resurgence of vitamin D deficiency rickets.

Before the industrial and agrarian revolutions, the technology of clothing and shelter dwelling enabled us to migrate mostly northward 60 k.y.a out of the tropics into regions of low levels of UVR. Whereas, biological adaptation through changes in gene frequency in a population or species and modification of population homozygosity takes hundreds of thousand years, cultural adaptation is rapid and depends on the charisma and authority of a few individuals. The more recently a group migrated into an area, the more extensive its cultural, but not biological, adaptation to the area will be (45).

Although the Sudanese and Saudis live at similar latitudes, Jablonski and Chaplin noted that the Sudanese have a dark skin color and the Saudis have a light skin color (45). The Sudanese have been dwelling at average latitude 13N ever since *H. sapiens* migrated throughout Africa and their skin color is well adjusted for high-intensity UVB radiation. Dwellers of the Arabian Peninsula have lived at similar latitude only since they arrived there from Europe 2,000 years ago. This time frame does not allow for biological adaptation, and they had utilized mostly cultural means to adapt to the same intensity of UVR. Specifically, they wear long protective clothes, they carry their shade with them in the form of tents, and they protected their lighter-skinned women using customary veils and house confinement. Traditional diets in indigenous people in northern latitudes included fatty fish and the blubber from seals and whales which also were sources of vitamin D. Hence, the circulating vitamin D levels in these people are high (74). Change in eating habits and reduced consumption of these traditional foods in populations living in the Arctic regions has been accompanied by a high prevalence of vitamin D deficiency in the last few decades (75, 76).

## Concluding Remarks and Future Perspectives

Sun exposure and skin photosynthesis are the major sources of vitamin D for both children and adults. Sensible sunshine exposure needs to consider all the above-mentioned benefits, but also the damaging effect of UVR on sunburn, DNA stability, folic acid levels, and skin cancer risk. In individuals with a dark complexion, the risk is small and benefits are great.

In the absence of the sun's UVR, it is difficult to obtain an adequate amount of vitamin D from nutritional sources without supplementation. The current pandemic of rickets can be attributed to our modern lifestyle in which outdoor activities are greatly reduced (6). This reduction reduces our exposure to the sun's UVB, which is required for the generation of vitamin D in the skin. It is unlikely that an individual who spends his/her entire time indoors would be exposed to the amount of UVR that is required to generate a sufficient amount of vitamin D as a result of human evolution. For contemporary individuals, vitamin D is an essential micronutrient and is no longer the sunshine vitamin because we now need to consume it in our diet. This is easy to prove: we use the plasma 25OHD concentration as a marker of supply, and we use the plasma concentration of parathyroid hormone and serum (77) and urinary phosphorus (78) as markers of function.

An Endocrine Society Clinical Practice Guideline recommends measuring vitamin D in individuals at risk for vitamin D deficiency and that infants and children aged 0–1 year need at least 400 IU/d of vitamin D and children 1 year and older require at least 600 IU/d to maximize bone health (79). These recommendations are in agreement with those from the European Society for Pediatric Endocrinology (80). Whether these doses of vitamin D are enough to provide these individuals with all the non-skeletal health benefits to maximize bone health and immune function is not known at this time (81). While the average serum 25OHD levels in contemporary adult hunter gatherers in East Africa are 46 ng/dL (115 nmol/l) (82), the guidelines recommend a blood level of 25OHD above 30 ng/ml (75 nmol/l), which may require at least 1,000 IU/day of vitamin D (79). We know of no disadvantage to increasing the vitamin D intake in children. Cases of vitamin D intoxication in individuals supplemented with megadoses of oral vitamin D are rare and usually asymptomatic.

Previous discussions on vitamin D deficiency were usually limited to mineral homeostasis and rachitic bone disease. Evidence is now plentiful that vitamin D has a multiplicity of non-calcemic functions, of which the most important in terms of natural selection is probably the regulation of innate immunity and the prevention of TB and possibly additional serious infections. Future studies need to include the effects on the innate immune system when assessing the outcomes of vitamin D supplementation and when treating nutritional rickets.

Dark pigmented individuals have a higher prevalence of vitamin D deficiency and secondary hyperparathyroidism. Although they have a higher average bone mineral density, are less prone to osteoporotic fractures (83–85) and may be more resistant to the bone-resorbing effect of PTH (86–88), there is no evidence that the optimal vitamin D levels needed for non-skeletal vitamin D functions are affected by skin pigmentation. Many of the benefits of vitamin D sufficiency where demonstrated in dark pigmented individuals and there is no data to support different vitamin D sufficiency cutoff for different populations. Due to their lower skin synthesis of vitamin D, dark skinned individuals in temperate zones most likely need higher doses of vitamin D supplementation all year round.

In some studies, body mass index and adiposity have been negatively correlated with the change in vitamin D status following vitamin D supplementation (89, 90). Obese children and adults require 2–3 times more vitamin D than non-obese children and adults to normalize circulating 25OHD levels. However, we do not know whether non-obese and obese children and adults require similar 25OHD levels for calcium balance and maintaining the integrity of other vitamin D-dependent functions. In an era of an obesity epidemic, we need more research to determine whether adiposity should be taken into account when determining the dietary requirements for vitamin D and calcium and the optimal serum 25OHD levels.

Finally, in an era of data technology, we need (a) to accumulate and analyse data to determine which individuals will get nutritional rickets, (b) to design effective preventive measures that can feasibly reach entire communities, and (c) apply preventive measures which may differ between various affected regions in darkly pigmented individuals living in temperate climates.

## Author Contributions

All authors listed have made a substantial, direct and intellectual contribution to the work, and approved it for publication.

### Conflict of Interest Statement

The authors declare that the research was conducted in the absence of any commercial or financial relationships that could be construed as a potential conflict of interest.

## GLOSSARY

**Pleistocene:** A geological epoch, which lasted from about 2,600,000 to 11,000 y.a., between the Pliocene and Holocene epochs. It covers most of the last glacier period.

**Holocene:** A geological epoch, which started after the last glacial period, 11,000 y.a. and ended recently with advent of the Anthropocene.

**Anthropocene:** A recently proposed geological epoch from the commencing of significant human impact on the Earth's geology and ecosystems, including anthropogenic climate change.

**Paleolithic:** Early Stone Age. An archeological period in human prehistory renowned by the earliest development of stone tools some 3.3 million y.a. to the end of the Pleistocene.

**Neolithic:** Late stone age. An archeological period in human history designated from the domestication of plants and animals 10-12 k.y.a.
